# Health Risks and Potential Predictors of Fatigue and Sleepiness in Airline Cabin Crew

**DOI:** 10.3390/ijerph18010013

**Published:** 2020-12-22

**Authors:** Candice C. Y. Wen, Christian L. Nicholas, Sandy Clarke-Errey, Mark E. Howard, John Trinder, Amy S. Jordan

**Affiliations:** 1 Melbourne School of Psychological Sciences, University of Melbourne, Parkville, VIC 3010, Australia; cln@unimelb.edu.au (C.L.N.); mark.howard@austin.org.au (M.E.H.); johnat@unimelb.edu.au (J.T.); ajordan@unimelb.edu.au (A.S.J.); 2 Institute for Breathing and Sleep, Austin Health, Heidelberg, VIC 3084, Australia; 3 Statistical Consulting Centre, University of Melbourne, Parkville, VIC 3010, Australia; sjclarke@unimelb.edu.au

**Keywords:** flight attendant, circadian disruption, sleepiness, fatigue, insomnia, shift work disorder, safety implications

## Abstract

Background: Aviation pilots and cabin crew regularly undertake shift work, and may experience circadian disruption, restricted sleep, sleepiness and impaired health. Research on aviation fatigue and sleepiness has focused on pilots, with less being known about cabin crew. This study aimed to identify likely predictors of fatigue, sleepiness, shift work disorder (SWD) and depression in cabin crew. Methods: An online anonymous survey was distributed to active cabin crew around the world. It measured sleepiness, fatigue, and screened for insomnia, depression and SWD. Information on individuals’ habits and work schedules were collected. Results: 930 valid responses were analysed. 63.5% of the sample had abnormal levels of fatigue and 46.9% experienced excessive daytime sleepiness. 68.0% were at risk for SWD, 57.7% screened positive for insomnia, and 40.0% for depression. Caffeine and use of alcohol and drugs for sleep were independently associated with insomnia and SWD (*p* < 0.05), whereas, type of route (international, domestic, both) and number of duty days per week predicted fatigue (*p* < 0.05). Conclusions: Cabin crew had a high prevalence of fatigue, sleepiness and elevated risk for SWD, insomnia and depression. Many cabin crew engaged in behaviours detrimental to good sleep hygiene, highlighting targets for future interventional studies.

## 1. Introduction

Prior to the COVID-19 pandemic, the aviation industry never rested. While it is unclear how long it will be until global travel returns to the pre-pandemic levels, it is expected that the aviation industry will resume similar operations in the years to come and the majority of aviation employees (e.g., pilots and cabin crew) will resume shift work. With the 24-h nature of shift work, a large portion of working hours frequently coincide with one’s biological sleep window. Therefore, the disruption of one’s circadian rhythm often results in individuals feeling sleepy while on the job (e.g., night shift), however unable to fall and/or maintain sleep after work (e.g., daytime). Excessive sleepiness at work has been associated with increased numbers of accidents and errors with an estimated cost of US $71 to 93 billion per year [1]. Past research has also found shift work contributes to health problems including insomnia [2], cardiovascular diseases [3], gastrointestinal complaints [4], higher body mass index [5,6], diabetes [7,8], cancer [9] and poorer mental health [10].

Extensive research indicates that fatigue and sleepiness have detrimental impacts on shift workers’ physical and mental health [2,11,12]. Fatigue is a feeling of exhaustion due to an accumulation of mental and/or physical exertion. Whereas the feeling of sleepiness is dependent on the length of time one has been awake, along with the timing of an individual’s internal circadian clock. Due to the heavy volume of flights traveling great distances across time zones, many pilots and cabin crew suffered from circadian disruption in addition to shift work. It is therefore not surprising that fatigue and sleepiness were common complaints in aviation employees [13,14,15,16].

Previous literature investigating fatigue and sleepiness in the aviation industry has focused heavily on airline pilots and found poor sleep quality, circadian misalignment and impaired cognitive performance [14,17,18]. However, research on pilots cannot be readily applied to cabin crew as the workforce demographic and type of work varies greatly between the two. For example, cabin crew are predominantly female whereas pilots are primarily male, and cabin crews’ workload is more physical compared to pilots’ higher mental workload. Beyond service delivery to passengers, cabin crew also perform critical safety roles during the normal course of a flight (e.g., safety briefings, aircraft security checks). These include responses to emergencies (e.g., fire on-board, emergency landing/ditching), and more commonly, first response when medical attention of a passenger is required. Studies estimates that prior to the COVID-19 pandemic, 44,000 inflight medical emergencies occurred around the world annually, which is equivalent to 1 medical emergency per 600 flights [19]; thus it is vital that cabin crew are vigilant while on duty. While the International Air Transport Association (IATA) stipulates that both pilots and cabin crew receive fatigue education as a component of their prescriptive safety training [20], the limited research on cabin crew has shown that, compared to pilots, cabin crew have a greater number of fatigue symptoms [21] and less sleep on off-duty days [22]. Therefore, understanding fatigue and sleepiness in cabin crew is important.

Cabin crew engage in complex work rosters (long, irregular work hours, frequently disrupted schedules) and are often required to sleep away from home; all of which increases the likelihood of being tired [23]. Prior research has found cabin crew underestimate their fatigue and sleepiness, and often start their workday not well rested [24,25]. With regards to fatigue, one study found 37% of cabin crew sought medical assistance due to experiencing frequent fatigue over the past year [26]. Fatigue in cabin crew has been found to negatively impact work performance; one study found 84% of crew reported being fatigued while on duty, and of that, 71% felt their safety-related performances were impaired [27]. Studies on sleepiness have shown that more than half of the cabin crew surveyed fell asleep during flights in the previous month [27], and reports of fatigue and sleepiness while driving home after a flight were common including several reports of falling asleep while driving [28]. The high rates of sleepiness may be related to difficulty in achieving consolidated sleep, likely a result of circadian disruption. Sleepiness may also result from failed preflight sleep due to an unfavorable circadian phase [29]. In addition, almost one third of the cabin crew were affected by sleep disorders [26]. In order to improve/promote sleep, cabin crew reported employing a number of different coping strategies including the use of drugs (anxiolytics and sleeping pills) and alcohol, or sometimes both [25,27,30,31]. How effective these strategies are at improving fatigue and sleepiness in cabin crew remains unclear, in part because prevalence rates of fatigue and sleepiness have not been firmly established. Fatigue and sleepiness pose significant risks to airlines operationally and cabin crew personally as sleep and fatigue are major factors contributing to cabin crew’s quality of life [32].

Mental health and wellbeing are also major concerns in the cabin crew community [33]. It has been estimated that by 2030, healthcare costs for mental disorders around the world would be US $16.3 million, with depression currently accounts for 4.3% of diseases [10]. The prevalence of depression amongst cabin crew is at least twice that of the general population in the United States [26], with up to 40% of active cabin crew having had a history of serious depression [34]. Past literature has associated depression with shift work disorder [35,36,37,38,39]. Thus, given the concerning incidence of depression amongst airline cabin crew, it is of importance to assess shift work disorder in this population. Shift work disorder (SWD) is classified as a circadian rhythm sleep disorder under the International Classification of Sleep Disorder (ICSD) [40]. Symptoms of SWD are excessive sleepiness at work when the work schedule overlaps one’s habitual sleeping time, and insomnia characteristics of difficulty initiating and/or maintaining sleeping when at home. The reported prevalence of SWD in other industries has varied between studies from 10–38% [35,41]. Some of this variance may be due to occupation type, with the risk of SWD varying from 15% among police officers [42] to 23% in oil rig workers [43] and 24–38% of nurses [36,44]. To our knowledge to date SWD has not been quantified in airline cabin crew.

Previous research has provided some insight into fatigue, sleepiness and mental health in cabin crew. However, no studies have been conducted to identify predictors of fatigue and sleepiness in airline cabin crew. Also, as SWD is a relatively new diagnosis, it would be beneficial to understand how susceptible cabin crew are to the disorder, and its association with depressive symptoms. Thus, the current study aimed to quantify fatigue and sleepiness, and to screen for insomnia, depression and shift work disorder in order to identify their potential predictors, such that future intervention studies to improve cabin crew health could be developed.

## 2. Materials and Methods

### 2.1. Participants

Currently active and full-time cabin crew from around the world were invited to participate in an anonymous online survey between February and May 2018. Participants were recruited using several methods. Firstly, the survey was advertised via The University of Melbourne (Australia)—Melbourne School of Psychological Sciences’ Facebook page for one week, targeting specific cabin crew groups. It was then allowed to snowball with increased ‘shares’ on the social media platform. The Facebook advertisement was deemed more effective when targeted at specific regions; thus, the advertisement was directed towards nations that host major airline hubs; the United States, United Kingdom, United Arab Emirates, Hong Kong, Singapore, Australia and New Zealand. Secondly, the National Division of Flight Attendant Association of Australia (FAAA) participated by emailing the survey link to its union members. Thirdly, the study was distributed via word of mouth from the researchers, using emails and sharing the Facebook advertisement. The study was approved by the Human Research Ethics Committee of The University of Melbourne, Ethics ID: 1750491.1. Participants provided digital informed consent.

### 2.2. Design

The survey was administered using QualtricsXM survey software (www.qualtrics.com) (Qualtrics, Seattle, WA, USA) and was designed to be accessible from computers and mobile devices. The survey was anonymous, with no request for participants’ identification.

The survey was designed to be completed in ten minutes, with mostly multiple-choice questions, and open questions confined to collection of demographic data. It collected both individual demographic and operational information. Individual variables included: age, gender, home travel time, mode of transport to and from work, caffeine consumption, alcohol and drugs usage. Operational information collected included: seniority, tenure, routes (international, domestic or both), work schedules detailed from the past month through to the participants’ latest trip (consecutive work days, sectors per day, average trip hours, duty days per week, estimated time of departure and estimated time of arrival), scheduled breaks and flight delays. The survey also included six validated scales as detailed below, to assess fatigue and sleepiness, as well as to screen for insomnia, shift work disorder and depression.

### 2.3. Scales to Measure Health Outcomes and Risks

#### 2.3.1. Fatigue

Subjective fatigue was measured using two scales: The Flinders Fatigue Scale (FFS) [45] and the Samn-Perelli Crew Status Check (SP) [46]. The FFS is a seven-item questionnaire which provides a definition of fatigue and distinguishes it from sleepiness (Cronbach’s alpha = 0.91). The FFS scores range from 0 to 31 and measures the experience of fatigue over the previous two weeks. The SP scale is commonly used in the aviation industry to measure fatigue. It is a 7-point scale from “1: Fully alert; wide awake, extremely energetic” to “7: Completely exhausted; unable to function effectively”. Individuals are asked to select the statement that best describes how they feel at a given point in time. In this survey, participants were asked to recall the level of fatigue based on their most recent trip; at the outbound sector flight’s take off and top-of-descent (when the aircraft is prepared to descend; normally thirty minutes prior to landing), the inbound sector take off and top-of-descent, and finally on the commute home.

#### 2.3.2. Sleepiness

Sleep Condition Indicator (SCI) [47] was used to measure sleep quality and to screen for insomnia. The SCI is an eight-item questionnaire based on the most recent month’s sleep; the individual scores range from 0 to 32, with higher scores indicating better sleep (Cronbach’s alpha = 0.86). SCI score ≤16 indicates possible insomnia disorder based on the DSM-5 criteria. Daytime sleepiness was measured using Epworth Sleepiness Scale (ESS) [48]. The ESS is a 24-point scale assessing one’s sleepiness in the past month. Participants rate sleepiness from 0–3 over eight activities (Cronbach’s alpha = 0.88).

#### 2.3.3. Shift Work Disorder and Depression

Screening for shift work disorder was achieved using the 4-item shift work disorder questionnaire (SWDQ; sensitivity = 0.74, specificity = 0.82) [49]. The questionnaire assessed sleepiness and well-being over the past month while working on non-standard shifts (starts before 7 am or after 2 pm, rotates, or regularly includes hours outside of the standard 7 am to 6 pm workday). If a participant responds ‘no’ to typically working a non-standard shift, the SWDQ was automatically omitted. Major depressive disorder was screened using the 2-item Patient Health Questionnaire (PHQ-2; sensitivity = 0.83, specificity = 0.92) [50]. The PHQ-2 asks about the frequency of depressed state and anhedonia over the past two weeks. PHQ score ≥3 indicates possible major depressive disorder.

#### 2.3.4. Safety

Perception of safety was measured in two ways. Firstly, cabin crew were asked to rate “how many times over the last fortnight did you feel your ability to perform safety-related tasks was compromised due to fatigue?”. The answers used a 5-point scale ranging from ‘never’ to ‘always’. Secondly, cabin crew were asked how frequently they fell asleep on the jumpseat during a critical phase (take off and/or landing) of flight. Falling asleep on the jumpseat during a critical phase is a serious offense because these are the periods when most major aviation incidents occur [51], thus cabin crew are trained to be alert and situationally aware for emergency scenarios during these times [52]. The answers ranged from a 5-point scale from ‘none’ to ‘five times and more’ over the last month.

### 2.4. Measures of Habits and Behaviours

Cabin crew were asked to provide the average intake of caffeine per 24-h period (coffee, coca cola, tea, hot chocolate and energy drinks included). The frequency of alcohol and drugs (over-the-counter, prescription, herbal and recreational) used to facilitate sleep was also collected. The answers ranged from a 5-point scale from ‘never’ to ‘always’.

### 2.5. Data Processing and Analysis

The FFS, ESS, SCI and PHQ-2 scores were obtained by summing the ratings for all the items in the scales and treated as continuous variables. The SWD is a dichotomous variable; yes or no to potential shift work disorder based on the results of the SWDQ. Each statement from the SP scales was handled individually, as it measured different phases of the work trip. Perceptions of safety and frequency of sleeping on the jumpseat were treated as continuous variables.

The remaining variables were divided into either ‘individual’ or ‘operational’ variables, as listed in the study design. Any insufficient responses per category were collapsed with a neighbouring response. Most of the individual and operational variables were treated as continuous variables apart from gender, route of flight, estimated time of departure (ETD) from outbound flight, estimated time of arrival (ETA) from inbound flight and mode of transport. These variables are categorical variables.

The mode of transport selected for home travel was re-categorised. Instead of type of vehicle used (e.g., personal vehicle, taxi, train etc.), it was categorised into ‘self-drive’, ‘picked-up’ and ‘public transport’. Picked-up included pick up from friends and family, taxi, and airline assigned transportation. Public transport included bus, boat/ferry, train, subway and plane. As respondents were able to select multiple modes of transport, separate dummy variables were created.

All statistical analyses were carried out using IBM Statistical Package for the Social Sciences (SPSS) version 25 (IBM, New York, NY, USA). Continuous variables are presented as mean ± standard deviation (mean ± SD) unless otherwise stated. The sample representativeness was assessed by comparing the gender and age with 2016 Australian Census travel attendants’ data using Pearson’s chi-square tests. The descriptive statistics of each scale and measures were assessed for frequency of each health outcome.

Correlation analysis was carried out to examine the relationships between the health outcomes, and individual and operational variables. Spearman’s correlation was carried out as the majority of variables were ordinal, with exception of two dichotomous variables; gender and SWD, where point biserial correlation was used. Only variables that significantly correlated with each scale were used in the subsequent regression analysis (significance cut-off was *p* < 0.05).

Multiple regression was used to identify which individual and/or operational variables predicted fatigue, sleepiness, depression and perception of safety. Logistic regression was conducted to predict shift work disorder as it was a dichotomous variable. Additional categorical variables were added directly to the regression analyses including; type of route (international, domestic or both), ETD, ETA and home transportation (self-drive, picked-up or public transport). Subsequent analysis of variance (ANOVA) were carried out for these variables if they were significant predictors. Regression analyses will be reported with beta value or odds ratio, 95% confidence interval and *p*-value; unless stated otherwise.

## 3. Results

A total of 1616 cabin crew consented to participate in the survey. Of these 496 were excluded due to incomplete surveys, with a further 190 excluded for not meeting the inclusion criteria of being currently active (*n* = 73) and full time (*n* = 117) cabin crew.

A total of 930 valid responses were used for data analysis. Participants’ demographic details are shown in Table 1. Data collected from incomplete surveys (*n* = 496) showed the average age of these respondents was 31.32 years, with a gender split of 23.2% male and 76.8% female; similar to the participants included in the sample group (Table 1). Australian based cabin crew had the greatest number of responses (42.5%), thus demographic comparison data was extracted from Australian references. The 2016 Australian Census showed that 73.6% of travel attendants identify as female, and 26.3% as male (note: travel attendants also included maritime and railway steward/ess). Chi-square goodness of fit analyses showed a non-significant difference for gender (X2 (1) = 0.54, *p* = 0.46) between the 2016 Australian census data and the current study sample. Average age for travel attendants in Australia is 33 years old [53], which was also consistent with the current sample. However, significant differences were found for age distribution with chi-square analyses (X2 (5) = 169.42, *p* < 0.01), indicating the current sample was skewed towards younger age compared to the Australian travel attendants’ population.

### 3.1. Health Outcomes and Risks

The occurrence of fatigue, sleepiness, insomnia, depression and shift work disorder are shown in Table 2. The cut-off scores for the relevant scales are detailed in the legend of Table 2. Cabin crew on average experienced borderline fatigue based on the Flinders Fatigue Scale (FFS). However, 12.4% of respondents indicated they had experienced severe fatigue (FFS ≥ 21) over the fortnight preceding completion of the survey. Results from the most recent trip using the SP scales (Table 2) showed that the majority of the cabin crew were not fatigued on take-off on the outbound sector, however, by top of descent, almost a third of the crew recalled being fatigued. Interestingly, on the inbound sector at take-off, despite 56.1% of crew having had some rest (e.g., layover at outstation, inflight crew rest), the fatigue (SP = 3.45) was only marginally lower than at the top of descent on the outbound sector (SP = 3.65) and was not back at the baseline fatigue level of outbound take-off (SP = 2.45). The SP mean for commute home was high, with more than three quarters of the cabin crew recalling being fatigued.

The cabin crew experienced mild sleepiness on average, however, 14.6% of the sample reported severe excessive sleepiness (ESS ≥ 16) in the past month. In addition, more than half of the sample were at risk for insomnia disorder, 40.0% of respondents were at high risk for major depressive disorders and two thirds of respondents were at high risk of shift work disorder.

Table 3 shows the frequency over the last fortnight that participants felt their ability to perform safety-related tasks was compromised due to fatigue. 78.2% reported they have experienced fatigue that may have affected their safety-related tasks at some point, with 20.4% experiencing this half of the time or more. 34.8% of the respondents fell asleep on the jumpseat during a critical phase over the last month, with 2.5% falling asleep in this situation five or more times within a month.

### 3.2. Habits and Behaviours

Table 4 shows the average intake of caffeine per day, and the use of alcohol and drugs (over-the-counter, prescription, herbal and recreational) to facilitate sleep. 91.1% of the sample consumed caffeinated drinks daily, with 9.9% consuming more than 5 servings per 24-h period.

Given the high occurrence of sleepiness and potential insomnia in this sample, it was of interest to examine the frequency of use of alcohol and drugs used to assist sleep. 41.4% of respondents reported using alcohol to facilitate sleep, with 5.7% using it most of the time, if not always. Use of drugs was less prevalent, with herbal drugs (e.g., valerian root) most commonly used, followed by over-the-counter drugs, and lastly, prescription drugs. Recreational drugs (e.g., marijuana) were almost never reported, therefore the variable ‘recreational drugs’ was removed for subsequent analyses.

The mode of transport used to and from work varied. 54.5% of the sample have used personal vehicles to commute to work, while 41.5% have received some form of pick-up (family and friends, taxi or airline provided transportation). 18.5% have utilised public transportation to commute between work and home.

### 3.3. Potential Predictors of Health Outcomes

Initial correlation analysis indicated age was strongly correlated with tenure for a participant’s current airline (r = 0.786, *p* < 0.01) and tenure over the lifetime (r= 0.841, *p* < 0.01), therefore the decision was made to remove both tenure variables. The total hours travelled for the most recent trip was strongly correlated to the number of days this trip took up (r = 0.793, *p* < 0.01), thus the number of trip days was also removed from further analyses. The remaining variables showed no evidence of any serious multicollinearity.

Table 5 shows the results of regression analyses predicting insomnia (SCI), sleepiness (ESS), depression (PHQ-2) and shift work disorder (SWDQ). Table 6 displays the regression results from fatigue and related outcomes; FFS, perception of safety, and Samn-Perelli measure of fatigue during the commute home (SP5).

#### 3.3.1. Insomnia and Sleepiness

Individual habits and behaviours were significant predictors of insomnia and quality of sleep (Table 5). For every unit (1–2 servings) increase in caffeine consumption per day, a decrease of 0.73 units in sleep quality score (95% CI: −1.35, −0.12, *p* = 0.02) was reported. Or alternatively, for every unit increase in caffeine consumption, insomnia increased by 0.73 units. SCI score was also associated with the use of alcohol (β: −0.73, 95% CI: −1.36, −0.11. *p* = 0.02) and drugs to assist sleep including over-the-counter (β: −0.89, 95% CI: −1.56, −0.21. *p* = 0.01) and herbal (β: −1.00, 95% CI: −1.61, −0.39. *p* < 0.01) medications. The duration of time to travel home significantly predicted sleep quality, with longer home travel time associated with poorer sleep quality/increased risk of insomnia (β: −0.65, 95% CI: −1.16, −0.13. *p* = 0.01). Type of route was also significantly associated with insomnia and sleep quality. When comparing type of routes (international, domestic, both), international cabin crew reported lower quality of sleep compared to domestic (β: −2.96, CI: −1.09, −4.82. *p* < 0.01) and both (β: −1.58, CI: −0.00, −3.16. *p* = 0.05) routes. The maximum number of consecutive days worked in the last fortnight was a predictor of excessive daytime sleepiness. Experiences of daytime sleepiness increased with higher number of consecutive works days (β: 0.29, 95% CI: 0.06, 0.52. *p* = 0.01).

#### 3.3.2. Depression and Shift Work Disorder

Correlation analyses showed shift work disorder and depression were significantly correlated (r = 0.26, *p* < 0.01), suggesting an association between these constructs which is consistent with past research [35,36,37,38,39].

Logistic regression was used to identify predicators for shift work disorder. For each one-point increase in the alcohol use scale, there was 1.26 times increase in the odds of shift work disorder (95% CI 1.02, 1.55. *p* = 0.03). The odds of shift work disorder also increased with every unit increase in the use of over-the-counter (OR: 1.47, 95% CI: 1.16, 1.87. *p* < 0.01), prescription (OR: 1.39, 95% CI: 1.01, 1.89. *p* = 0.04) and herbal (OR: 1.37, 95% CI: 1.11, 1.70. *p* < 0.01) drugs to aid sleep. The use of public transport to get to and from work decreased the odds of shift work disorder (OR: 0.64, 95% CI: 0.44, 0.95. *p* = 0.03).

Depression was significantly associated with two individual variables, age (β: −0.02, CI: −0.03, −0.00. *p* = 0.01) and the use of alcohol to facilitate sleep (β: 0.18, CI: 0.02, 0.34. *p* = 0.03). Interestingly, age had a negative relationship with depression; for every year increase in age, a lowering of 0.02 units in PHQ-2 score was found, thus being older is associated with a reduction in the likelihood of major depressive disorder in this sample. The number of delayed flights in the last month significantly predicated depression along with type of routes (international, domestic or both). ANOVA was conducted to determine if depression levels differed between the typical routes cabin crew flew. Results indicate there was a significant effect of route [Welch’s *F*(2, 453.272) = 8.811. *p* < 0.01]. PHQ-2 score increased from domestic cabin crew (2.01 ± 1.74) to crew who operates both international and domestic routes (2.23 ± 1.59) and finally international cabin crew (2.58 ± 1.68). Games-Howell post hoc analysis revealed international cabin crew had greater risk for depression when compared to domestic cabin crew (*p* < 0.01), and crew who operate both international and domestic routes (*p* = 0.01).

#### 3.3.3. Fatigue

Overall, fatigue and related measures were predicted mostly by operational variables. FFS was significantly predicted by the average number of duty days per week (β: 0.61, CI: 0.21, 1.00. *p* < 0.01) and type of flight routes (international, domestic, or both). ANOVA was also conducted to determine if fatigue levels differed depending on the normal routes cabin crew flew. There was a significant effect of route [Welch’s *F*(2, 475.028) = 19.36. *p* < 0.01]. FFS scored lowest in domestic cabin crew (12.76 ± 4.68) compared to crew who operates both international and domestic routes (14.30 ± 4.75) and highest in international cabin crew (15.36 ± 5.24). Games-Howell post hoc analysis showed international cabin crew were more fatigued compared to domestic cabin crew (*p* < 0.01), and crew who operate both international and domestic routes (*p* = 0.01).

The perception of safety being compromised by fatigue was predicted by the average duty day per week (β: 0.09, CI: 0.02, 0.15. *p* = 0.01) and the number of delayed flights over the past fortnight (β: 0.07, CI: 0.02, 0.12. *p* < 0.01). The Samn-Perelli measure of fatigue on commute home (SP5) for the most recent trip showed associations with both individual and operational variables. Fatigue on the commute home increased with longer home travel time (β: 0.27, CI: 0.17, 0.36. *p* < 0.01) and greater consumption of caffeinated beverages in the past 24 h (β: 0.15, CI: 0.03, 0.27. *p* = 0.01). Interestingly, compared to other modes of transportation (self-drive, public transport), those getting picked-up to and from work reported increased fatigue on the commute home (β: 0.28, CI: 0.02, 0.54. *p* = 0.03). Also unexpectedly, receiving scheduled breaks during the most recent trip was a significant predictor for SP5 (β: 0.14, CI: 0.27, 0.01. *p* = 0.03); whereby, receiving more breaks during the most recent trip was associated with greater fatigue recalls during commute home. The number of sectors flown per day was associated with SP5, alongside estimated time of arrival for the most recent inbound flight.

The estimated time of arrival (ETA) for the most recent inbound flight (flight returning to base) was divided into six 4-h groups, around the clock. ANOVA analysis indicated a significant effect for the time of arrival (Welch’s *F*(5, 332.056) = 6.69. *p* < 0.01). The mean SP5 score was the lowest for flights with ETA between 16:00–19:59 h (4.97 ± 1.40), and the highest for flights ETA between 04:00–07:59 h (5.64 ± 1.15). Games-Howell post hoc analysis revealed cabin crew of flights returning to base between 04:00–07:59 h were more fatigued when commuting home compared to those with an arriving time between 16:00–19:59 h (*p* < 0.01).

## 4. Discussion

The results of this study indicate that a large proportion of this cabin crew population studied before the onset of the COVID-19 pandemic, experienced fatigue and sleepiness. From the initial screening of health risks, two thirds of the sample were at risk of shift work disorder, more than half were at risk for insomnia and finally more than one third at risk for major depressive disorder. The rate of insomnia, SWD and depression were predicted by variables related to the individual crew member (e.g., consumption of caffeine, use of alcohol and drugs for sleep). In comparison, fatigue measures were associated mostly with operational variables such as flight delays and rostering, which is understandable, as fatigue is an outcome of exertion.

The occurrence of SWD was high amongst this cabin crew population and was also high compared to previous studies in other shift work industries [36,42,43,44]. This may be influenced by the female predominance in airline cabin crew, while previous prevalence studies in policemen and oil rig workers have involved male dominant industries. Women in general report greater social and familial commitments and domestic burdens, therefore may have less allowance for shift work recovery compared to men [33]. However, compared to nurses also a predominantly female profession, airline cabin crew still had high rates of shift work disorder. This may potentially be due to the nature of shift work in the aviation industry which results in regular rapid shifting of desynchrony between the circadian rhythm and sleep/wake behaviours along with unpredictable changes due to factors such as delayed flights.

With 40% of the sample population at risk for depression, this almost mirrors past statistics on cabin crew [34]. Depression was negatively associated with age, with the younger cabin crew at greater risk for depression than older cabin crew. This is possibly due to a ‘survivor effect’, whereby those affected by depression would leave the job, thus the unaffected ongoing cabin crew continues in the job. As age was strongly correlated with tenures, this can also be linked with past research which found within airline seniorities, junior crew were more vulnerable to fatigue [24,57] and sleepiness; and had lower quality of life [32] compared to senior cabin crew.

Individual consumption of caffeine was a predictor of insomnia and sleep quality. Almost all the sample population consumed caffeinated beverages on a daily basis, with ten percent consuming five or more servings per day. This suggests cabin crew are using caffeine to combat sleepiness, however inappropriate timing of caffeine or excessive use may set off a vicious cycle of over stimulation, leading to difficulty initiating sleep when needed. Similar to previous research on cabin crew [25,27,30,31], the use of alcohol and/or drugs were strategies used to facilitate sleep and, in this study, they were associated with both insomnia and shift work disorder. Past studies found alcohol can exacerbate insomnia; while alcohol can facilitate sleep onset, it significantly disrupts sleep over all [58,59]. However, whether the drugs were: (1) taken incorrectly; or (2) not at the appropriate time/dose and therefore had counterintuitive effects on sleep; or (3) whether those with more severe insomnia and shift work disorder used more strategies to improve sleep, can’t be determined from this cross-sectional study. Alcohol was the substance most frequently used, followed by herbal drugs, then over-the-counter and finally prescription medication. The choice of alcohol and particular drugs may relate to their accessibility, especially when travelling away from home [31].

International cabin crew reported greater fatigue and higher risk for major depressive disorder compared to cabin crew who operated other types of routes (domestic or both). The fatigue findings are consistent with some past research which found long-haul or international routes were more taxing due to length of duty as well as time zone changes, compared to short-haul or domestic flights [60,61]. The majority of the cabin crew were not fatigued on take-off on the outbound sector from their most recent trip. Interestingly, on the inbound sector at take-off, despite more than half of crew having had some rest (e.g., layover at outstation, inflight crew rest), the fatigue was not back at the baseline fatigue level of outbound take-off. This suggests that rests outside of the comfort of one’s home, be it due to environmental factors (e.g., unfamiliar environment) or to sleeping at inappropriate circadian phase, are likely not to be as restorative or are of insufficient duration [25]. The SP mean for commute home (SP5) was high. This is alarming as a large proportion of the cabin crew reported driving home after flights. Past research found cabin crew were fatigued and sleepy while driving home [28]; however, the results from this study showed that it was the cabin crew who were picked up by someone else to and from work that reported greatest fatigue for their commute home when compared to other modes of transportation (self-drive, public transport). This suggests cabin crew may possibly take countermeasures to combat fatigue if they knew they had to drive home after flight (e.g., greater caffeine intake), or deliberately chose not to drive in anticipation of fatigue.

The study found inbound flights arriving between 1600–1959 h had the lowest level of reported fatigue for the commute home. This makes sense if an individual’s circadian rhythm has not been altered substantially during the trip. Thus, the cabin crew would have been operating the flight during the ‘normal’ waking hours as compared to an ETA of 0400–0759 h, whereby the cabin crew would have been working throughout the night, during the typical sleeping hours. Past research on pilot fatigue had similar findings with domicile overnight flights associated with reduced subjective alertness [62].

### 4.1. Future Directions

The current study suggests an intervention program aimed at lowering the high rates of fatigue, sleepiness and health problems experienced by cabin crew may be beneficial if/when airlines resume typical operations. Specifically, a behavioural/psychological program consisting of sleep hygiene education, scheduled sleep and napping, the sleep environment in-flight and optimising work periods in-flight to improve sleep. However, based on our results, we believe that sleep hygiene program should be central to the education program, thus building awareness on the use and effects of caffeine, alcohol and drugs to facilitate sleep. Sleep hygiene also promotes sleep strategies that can be used at home and on layovers, without alcohol or other pharmaceutical aids. Recent research has found an association between poor sleep hygiene and SWD among nurses, therefore a successful intervention could potentially result in multiple benefits [63]. We would also recommend cognitive behavioural therapy for insomnia (CBTi), or at least components of CBTi, for cabin crew who screen positive for insomnia. These could be delivered digitally allowing cabin crew to access the program whenever and wherever. Additional cognitive behavioural therapy components to address depression would likely be beneficial for many cabin crew also.

Further, when airlines resume operations in the post-pandemic era, they could consider modifying operational variables to improve fatigue and sleepiness, in particular for cabin crew operating international routes. The number of consecutive workdays was associated with increased daytime sleepiness. With increased number of duty days rostered in a week, being associated with greater levels of fatigue and a rise in perception of safety being compromised due to fatigue. Research in the healthcare industry has found that reducing the number of shifts and/or consecutive work hours for doctors improves sleep duration [64] and significantly lowers the number of medical errors [65]. The aviation industry may apply similar changes, such as introducing more rest days between trips to reduce fatigue and sleepiness. Both the number of flight delays and the duration of these delays were associated with insomnia, depression, fatigue and perception of safety. Not only are flight delays costly and counterproductive for airline operation, it also negatively impacts on the well-being of cabin crew. Thus, it would be a win-win situation for airlines to enforce strict action plans to minimise the number of flight delays where possible, and if not, reduce the duration of delayed flight. Likewise, flights arriving back to base between 0400 and 0759 were associated with increased fatigue on their commute home. Past research has found pilots frequently engaged in alert management strategies (e.g., strategic napping) during early morning hours [62], however, this may not always be possible with the service nature of cabin crew’s work. Therefore, minimising flights landing during this window may reduce working during unfavourable circadian phase and potentially increase alertness on the commute home.

### 4.2. Study Limitations

Despite its strengths of having a large sample size from around the world and using validated measures, the present study was not without limitations. Firstly, there is likely sample selection bias as a result of the snowball self-selection process as well as the fact that the title of the advertisement, was “Airline Cabin Crew: A Tiredness Study” due to Facebook advertisement restrictions on issues surrounding ‘well-being’. Thus, we may have attracted a more ‘tired’ cabin crew population than the general cabin crew population. It is worthy to note however, that there is clearly a sector of the population that requires assistance and will benefit with some form of intervention. The study was also likely to be more accessible to those with social media accounts and who are comfortable with digital survey. The second limitation was the use of screenings tools; PHQ-2 was the reduced versions of Patient Health Questionnaires for depression, it was chosen to maintain short participation time, and SWDQ was the only validated and appropriate SWD screening tool for this study, however it is based on the ICSD-2 criteria instead of the updated ICSD-3. Both of these tools were used for screenings purposes only. Future research should consider the use of more comprehensive diagnostic tools as well as to add measures such as chronotypes to evaluate its relationship with this shift work population and other sleep hygiene measures such as screen use before bedtime. Thirdly, with the nature of the study being cross-sectional, this provided a valuable snapshot of the current state, however its impact may be limited. Future research should consider a follow-up/longitudinal study to determine the strengths of the potential predictors. Finally, all measures were subjective. Future research could use actigraphy and work diaries, having both objective and subjective measures of fatigue and sleep would be of use, given that past research has found objective measures of sleep and fatigue were considerably worse than one’s own perception [24,61].

## 5. Conclusions

Prior to the COVID-19 pandemic, fatigue and sleepiness impacted a large proportion of airline cabin crew. Between 40–68% of the sample population screened positive for insomnia, depression, shift work disorder or a multitude of these disorders. Consumption of caffeine and use of alcohol and drugs to promote sleep was associated with increased levels of insomnia and reduced sleep quality. Fatigue levels impacted international cabin crew the most and was also associated with work rosters; number of sectors flown per day and number of duty days per week. An intervention program to reduce the impact of shift work in this population could improve mental health and safety for airline cabin crew. As the airlines industry recovers from the pandemic, they have an opportunity to design rosters and flight schedules to minimise fatigue and sleepiness for airline cabin crew which is likely to have significant positive health and wellbeing outcomes.

## Figures and Tables

**Table 1:** Cabin Crew demographic (*n* = 930).

Demographic	*n*	%
Age		
Mean ± SD: 32.7 ± 10.6 years	Range (19–63)	
Gender		
Female	695	74.7%
Male	235	25.3%
Route		
International	474	51.0%
Domestic	185	19.9%
Both	271	29.1%
Tenure (All airlines worked)		
Mean ± SD: 8.6 ± 8.2 years	Range (0–30)	
Seniority *		
Bottom one-third	405	43.5%
Middle one-third	302	32.5%
Top one-third	223	24.0%
Base		
Australia & New Zealand	477	51.3%
Middle East	205	22.0%
Europe	107	11.5%
Asia	101	10.9%
United States	37	4.0%
Anonymous	3	0.3%

* Bottom one-third: Economy Class cabin crew. Middle one-third: Business/First Class Crew. Top one-third: Purser/Management Crew.

**Table 2:** Occurrence of fatigue, sleepiness, insomnia, depression and shift work disorder (*n* = 930).

Measures	Scales	Mean ± SD	% at Risk or at Abnormal Level
Fatigue	Flinders Fatigue Scale (FFS)	14.53 ± 5.09	63.5%
	Samn-Perelli Crew Status Check (SP)		
	Outbound sector–take off	2.45 ± 1.32	8.7%
	Outbound sector–Top of descent	3.65 ± 1.48	31.6%
	Inbound sector–take off	3.45 ± 1.43	25.3%
	Inbound sector–Top of Descent	4.55 ± 1.42	58.2%
	Commute home	5.28 ± 1.30	77.4%
Sleepiness	Epworth Sleepiness Scale (ESS)	10.42 ± 4.58	46.9%
Insomnia	Sleep Condition Indicator (SCI)	15.54 ± 6.79	57.7%
Depression	Patient Health Questionnaire (PHQ-2)	2.36 ± 1.68	40.0%
Shift Work Disorder	Shift Work Disorder (SWDQ)	Positive for Shift work disorder: 68.0%	

Cut-off scores for the following scales: FFS [54]: ≤12 (normal), 13–15 (borderline fatigue), 16–20 (moderate fatigue) and ≥21 (severe fatigue); SP [55]: ≥5 (indicate excessive fatigue); ESS [56]: ≤10 (normal), 11–12 (mild sleepiness), 13–15 (moderate sleepiness), 16–24 (severe excessive sleepiness); SCI [47]: ≤16 screens possible insomnia disorder; PHQ-2 [50]: ≥3 screens for major depressive disorder.

**Table 3:** Frequency of safety related tasks being compromised by fatigue, and how many times crew fell asleep on the jumpseat (Total *n* = 930).

**Safety**	**Never**	**Sometimes**	**Half the time**	**Most of the time & Always**
	21.8%	57.7%	14.4%	6.0%
**Jumpseat**	**None**	**Once or twice**	**Three or four times**	**Five times and more**
	65.2%	24.8%	7.5%	2.5%

**Table 4:** Frequency of daily caffeine intake and use of alcohol and drugs to facilitate sleep (Total *n* = 930).

	None	1–2	3–4	5 or More
**Caffeine**	9.9%	46.6%	33.7%	9.9%
	**Never**	**Sometimes**	**Half the Time**	**Most of the Time & Always**
**Alcohol**	58.6%	31.2%	4.5%	5.7%
**Over-the-counter drug**	61.5%	26.5%	6.8%	5.3%
**Prescription drug**	80.6%	13.9%	2.9%	2.6%
**Herbal drug**	57.5%	31.6%	5.5%	5.3%
**Recreational**	98.0%	1.7%	0.0%	0.3%

**Table 5:** Summary of significant beta values and odds ratios for regression analysis predicting insomnia & sleep quality, sleepiness, depression and shift work disorder.

Variables	Insomnia & Sleep Quality	Sleepiness	Depression	Shift Work Disorder
Individual				
Age			−0.02 **	
Caffeine	−0.73 *			
Alcohol	−0.74 *		0.18 *	1.26 *
Over-the-counter drug	−0.89 **			1.47 **
Prescription drug				1.39 *
Herbal drug	−1.00 **			1.38 **
Home travel time	−0.65 *			
Public transport use				0.64 *
Operational				
Consecutive workdays		0.29 *		
Number of delayed flights	−0.78 **		0.19 **	
International vs Domestic	−2.96 **		0.81 **	
International vs Both	−1.58 *		0.49 *	

** *p* < 0.01; * *p* < 0.05. Only the variables that significantly predicted the outcome variables are presented.

**Table 6:** Summary of beta values on fatigue, perception of safety and fatigue during commute home.

Variables	Fatigue	Perception of Safety	Fatigue During Commute Home
Individual			
Home travel time			0.27 **
Caffeine			0.15 *
Picked-up			0.28 *
Operational			
Sectors/day			0.13 *
Duty days/week	0.61 **	0.09 **	
Breaks			0.14 *
Number of delayed flights		0.07 **	
Delay time average			0.18 **
International vs. Domestic	3.38 **		
International vs. Both	2.19 **		

** *p* < 0.01; * *p* < 0.05; Only the variables that significantly predicted the outcome variables are presented.

## Data Availability

The data presented in this study are available on request from the corresponding author.
